# Rapid detection of bovine rotavirus a by isothermal reverse transcription recombinase polymerase amplification assays

**DOI:** 10.1186/s12917-022-03437-8

**Published:** 2022-09-08

**Authors:** Yuelin Liu, Libing Liu, Jinfeng Wang, Xiaoxia Sun, Yaxin Gao, Wanzhe Yuan, Jianchang Wang, Ruiwen Li

**Affiliations:** 1 College of Veterinary Medicine, Hebei Agricultural University, No.2596 Lekai South Street, Baoding, Hebei 071001 People’s Republic of China; 2 Technology Center of Shijiazhuang Customs District, No.318 Heping Xi Lu, Shijiazhuang, 050051 People’s Republic of China

**Keywords:** BRVA, VP6 gene, Real-time RT-RPA, LFS RT-RPA, Isothermal amplification

## Abstract

**Background:**

Bovine rotavirus A (BRVA) is considered to be the most common pathogen of severe diarrhea in cattle worldwide, which could lead to the death of newborn calves and cause the significant economic losses to the cattle industry. As a novel isothermal nucleic acid amplification technique, recombinase polymerase amplification (RPA) has been applied widely for the rapid detection of different important pathogens in human and animals.

**Results:**

An RT-RPA assay based on the real time fluorescence monitoring (real-time RT-RPA) and an RT-RPA assay combined with a lateral flow strip (LFS RT-RPA) were successfully developed by targeting the VP6 gene of BRVA. The RT-RPA assays allowed the exponential amplification of the target fragment in 20 min. After incubation of the LFS RT-RPA on a metal bath at 40 °C, the results were displayed on the lateral flow strip within 5 min, while real-time RT-RPA allowed the real-time observation of the results in Genie III at 42 °C. Both of the two assays showed high specificity for BRVA without any cross-reaction with the other tested pathogens causing diarrhea in cattle. With the standard RNA of BRVA serving as a template, the limit of detection for real-time RT-RPA and LFS RT-RPA were 1.4 × 10^2^ copies per reaction and 1.4 × 10^1^ copies per reaction, respectively. In the 134 fecal samples collected from cattle with diarrhea, the BRVA positive rate were 45.52% (61/134) and 46.27% (62/134) in real-time RT-RPA and LFS RT-RPA, respectively. Compared to a previously published real-time PCR, the real-time RT-RPA and LFS RT-RPA showed a diagnostic specificity of 100%, diagnostic sensitivity of 98.39% and 100%, and a kappa coefficient of 0.985 and 1.0, respectively.

**Conclusions:**

In this study, BRVA was successfully detected in cattle fecal samples by the developed real-time RT-RPA and LFS RT-RPA assays. The developed RT-RPA assays had great potential for the rapid detection of BRVA in under-equipped diagnostic laboratory and the point-of-need diagnosis at quarantine stations and farms, which is of great importance to control BRVA-associated diarrhea in cattle herds.

## Background

Bovine rotavirus (BRV) belongs to the genus Rotavirus of the family Reoviridae [1], and was firstly identified as the cause of diarrhea in newborn calves in 1968 [2]. The genus Rotavirus has been divided into at least 10 different serogroups (A-J) [3], in which rotavirus A (RVA) is genetically and antigenically diverse [4] and also is the most common in the clinical findings [5]. Moreover, RVAs are the most widespread concern due to its ability to cause diarrhea in humans and a wide range of animals [6, 7]. Bovine rotavirus A (BRVA) is considered to be the most common pathogen of severe diarrhea in cattle, which could lead to the death of newborn calves and cause the significant economic losses to the cattle industry [8, 9]. BRVA is susceptible to newborn calves and causes severe clinical symptoms, mainly including the depression, loss of appetite, watery diarrhea and severe dehydration, even bloody stool discharge. Infected calves are susceptible to the secondary infections, which aggravates the disease and increases mortality. BRVA infection is prevalent worldwide and poses a serious threat to the cattle industry worldwide [9–12]. Furthermore, the genotyping and phylogenetic analysis demonstrated that the human RVA strains showed a close genetic relationship to the animal RVA strains [13]. It had been reported that BRVA could pose a threat to human health [1, 7]. Considering the economic losses to the cattle industry and the potential public health significance, the timely surveillance and detection of BRVA infection in cattle should be strengthened.

The establishment of rapid, specific and sensitive detection methods specific for BRVA is one of the effective means to prevent and control BRVA infection. The traditional virus isolation and identification [14] and serological diagnosis [15] were time-consuming and laborious, and could not meet the needs of rapid clinical diagnosis. A number of molecular biology assays based on PCR technology [16–18] had been widely used for BRVA detection, which demonstrated high detection efficiency. But these assays required high-precision and expensive instruments, professional technicians and good laboratory environment, which were not available in most laboratories with insufficient equipments or in field. The nucleic acid isothermal amplification techniques compensate for the difficulties of variable temperature amplification of PCR techniques. The developed reverse transcription loop-mediated isothermal amplification (RT-LAMP) assay specific for BRVA could amplify the target fragment at a constant temperature of 63 °C [19]. However, it had a long amplification time (60 min) and uses six primers in a reaction which made their design slightly more complex.

Recombinase polymerase amplification (RPA) is a novel isothermal amplification technique that mainly relies on three core enzymes to amplify target gene fragment, which are recombinase, single-stranded DNA-binding protein (SSB) and strand-displacement DNA polymerase [20]. RPA departs from expensive thermal cycling experimental apparatus and enables exponential amplification of target fragments in a short time at constant temperature, making it possible to perform pathogen detection under a variety of non-laboratory conditions [21]. Compared with other detection methods, the RPA method has the advantages of simple instrumentation, easy operation and rapid to obtain results. In this study, we designed specific primers, exo probe and nfo probe based on the conserved sequence of BRVA VP6 gene, established the real-time reverse transcription RPA (real-time RT-RPA) and RT-RPA combined with lateral flow strip (LFS RT-RPA) assays for the rapid, simple, and reliable detection of BRVA in the cattle fecal samples.

## Results

### Analytical specificity and sensitivity of the real-time RT-RPA assay

**Fig. 1:**
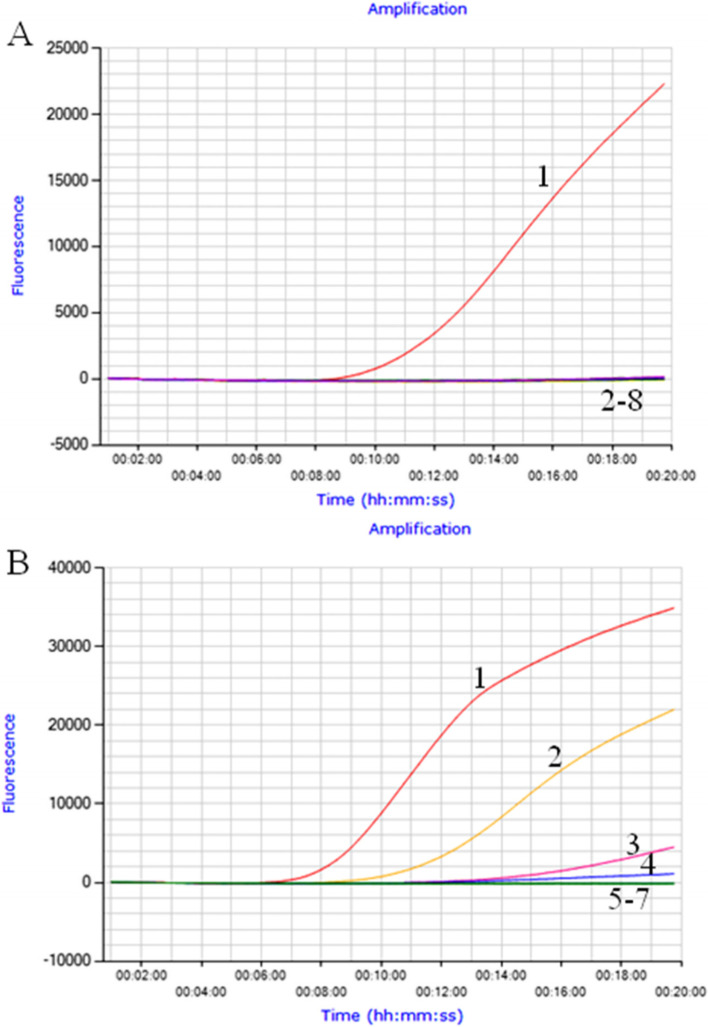
Performance of BRVA-specific real-time RT-RPA assay. Only the genomic RNA of BRVA was amplified, and the limit of detection of the assay was 1.4 × 10^2^ copies. **A**. Analytical specificity of the real-time RT-RPA assay evaluating on the common pathogens causing diarrhea in cattle. Line 1, BRVA; line 2, BVDV; line 3, BCoV; line 4, BEV; line 5, BKV; line 6, ETEC; line 7, *C*. *perfringens*; line 8, Nuclease free water. **B**. Analytical sensitivity of the real-time RT-RPA assay using a dilution range of 1.4 × 10^5^ ~ 1.4 × 10^0^ copies of BRVA standard RNA. Line 1, 1.4 × 10^5^ copies; line 2, 1.4 × 10^4^ copies; line 3, 1.4 × 10^3^ copies; line 4, 1.4 × 10^2^ copies; line 5, 1.4 × 10^1^ copies; line 6, 1.4 × 10^0^ copies; line 7, Nuclease free water

In the analytical specificity analysis, only the BRVA but not other pathogens and the non-template control (Nuclease free water) used in this study was detected (Fig. 1A). The same results were obtained in 3 repeats, which demonstrated the good specificity and repeatability of the developed real-time RT-RPA assay for BRVA.

In the analytical sensitivity analysis, 1.4 × 10^5^ ~ 1.4 × 10^2^ copies of BRVA standard RNA were detected in all the 8 runs, and 1.4 × 10^1^ ~ 1.4 × 10^0^ copies and the non-template control (Nuclease free water) were not detected in any run (Fig. 1B). Based on the above results, the limit of detection of the BRVA real-time RT-RPA assay was determined to be 1.4 × 10^2^ copies/reaction.

### Optimization of incubation temperature and time of the LFS RT-RPA

**Fig. 2:**
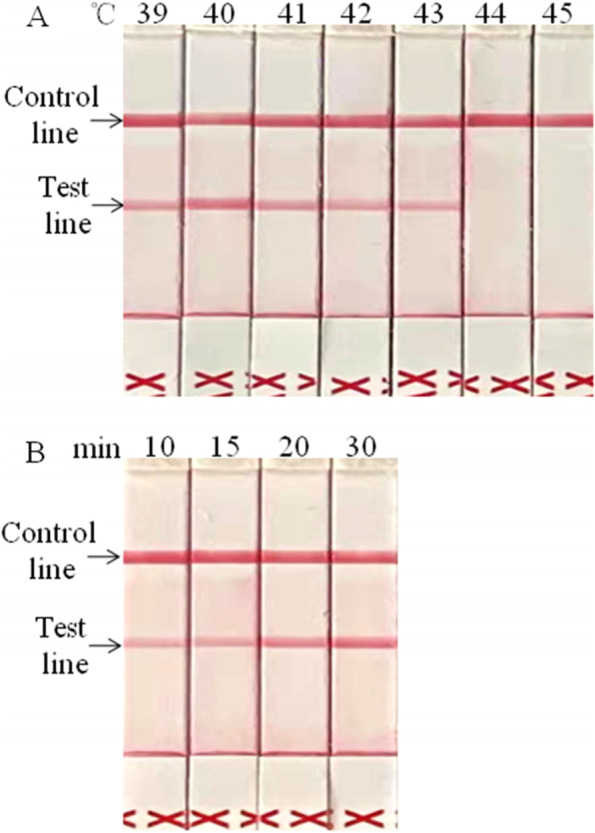
Optimization of the BRVA-specific LFS RT-RPA assay. The optimal reaction condition was set to be performed at 40 °C for 20 min. **A**. Optimization of the reaction temperature. The target gene of BRAV was amplified successfully at 39 °C ~ 43 °C. The red bands (test lines) were visible at 39 °C ~ 43 °C, and the test line band was clearest at 40 °C, which was set as the optimal reaction temperature. **B**. Optimization of the reaction time. The target gene of BRVA was amplified successfully at 40 °C for 10 min ~ 30 min. The red bands (test lines) were clearer after incubation for 20 min and 30 min, and there were no clear differences for them. The optimal reaction time was set as 20 min

The developed BRVA LFS RT-RPA worked successfully at 39 °C ~ 43 °C (Fig. 2A). The red bands (test lines) were visible at 39 °C ~ 43 °C, in which the test band was clearest at 40 °C. The same results were obtained in the parallel and the optimal reaction temperature was determined to be 40 °C. The developed BRVA LFS RT-RPA worked successfully at 40 °C after incubation of 10 min ~ 30 min (Fig. 2B). A weak red band on the test line was observed after 10 min, and it became clearer as the incubation time increased. There was no distinct difference between the red bands after incubation of 20 and 30 min. The same results were obtained from 3 repeats and the optimal reaction time was determined to be 20 min.

### Analytical specificity and sensitivity of the LFS RT-RPA Assay

**Fig. 3:**
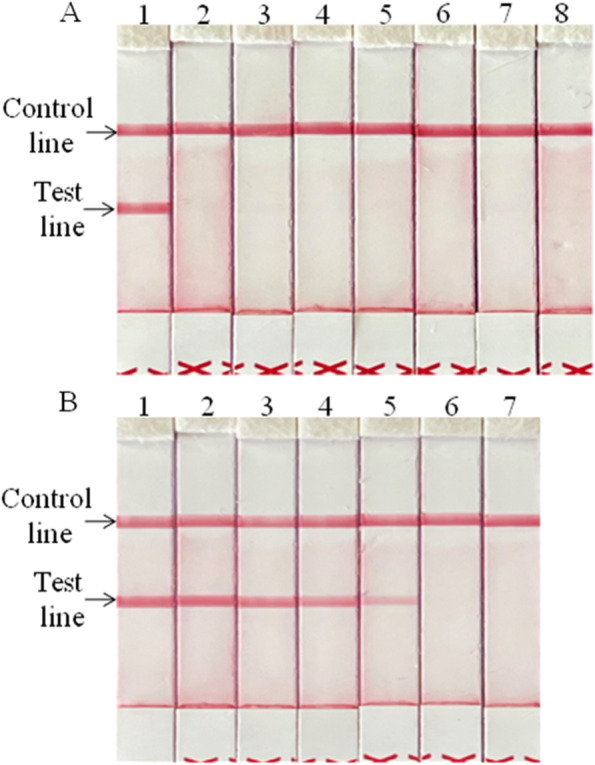
Performance of BRVA-specific LFS RT-RPA assay. Only the genomic RNA of BRVA was amplified, and the limit of detection of the assay was 1.4 × 10^1^ copies. **A**. Analytical specificity of the LFS RT-RPA assay evaluating on the common pathogens causing diarrhea in cattle. Line 1, BRVA; line 2, BVDV; line 3, BCoV; line 4, BEV; line 5, BKV; line 6, ETEC; line 7, *C*. *perfringens*; line 8, Nuclease free water. **B**. Analytical sensitivity of the LFS RT-RPA assay using a dilution range of 1.4 × 10^5^ ~ 1.4 × 10^0^ copies of BRVA standard RNA. Line 1, 1.4 × 10^5^ copies; line 2, 1.4 × 10^4^ copies; line 3, 1.4 × 10^3^ copies; line 4, 1.4 × 10^2^ copies; line 5, 1.4 × 10^1^ copies; line 6, 1.4 × 10^0^ copies; line 7, Nuclease free water

In the analytical specificity analysis, the red band was only observed in the test line when the BRVA RNA was used as the template but not other pathogens and the non-template control (Nuclease free water) used in this study (Fig. 3A). The same results were observed in 3 independent reactions, which demonstrated the good specificity and repeatability of the developed LFS RT-RPA assay.

In the analytical sensitivity analysis, the red bands could be observed in the test lines with 1.4 × 10^5^ ~ 1.4 × 10^1^ copies of standard RNA, and 1.4 × 10^0^ copies and the non-template control (Nuclease free water) were not detected in any run (Fig. 3B). All 5 independent reactions showed the same results, and the limit of detection of the BRVA LFS RT-RPA was determined to be 1.4 × 10^1^ copies/reaction.

### Validation of the developed assays on the cattle fecal samples

**Fig. 4:**
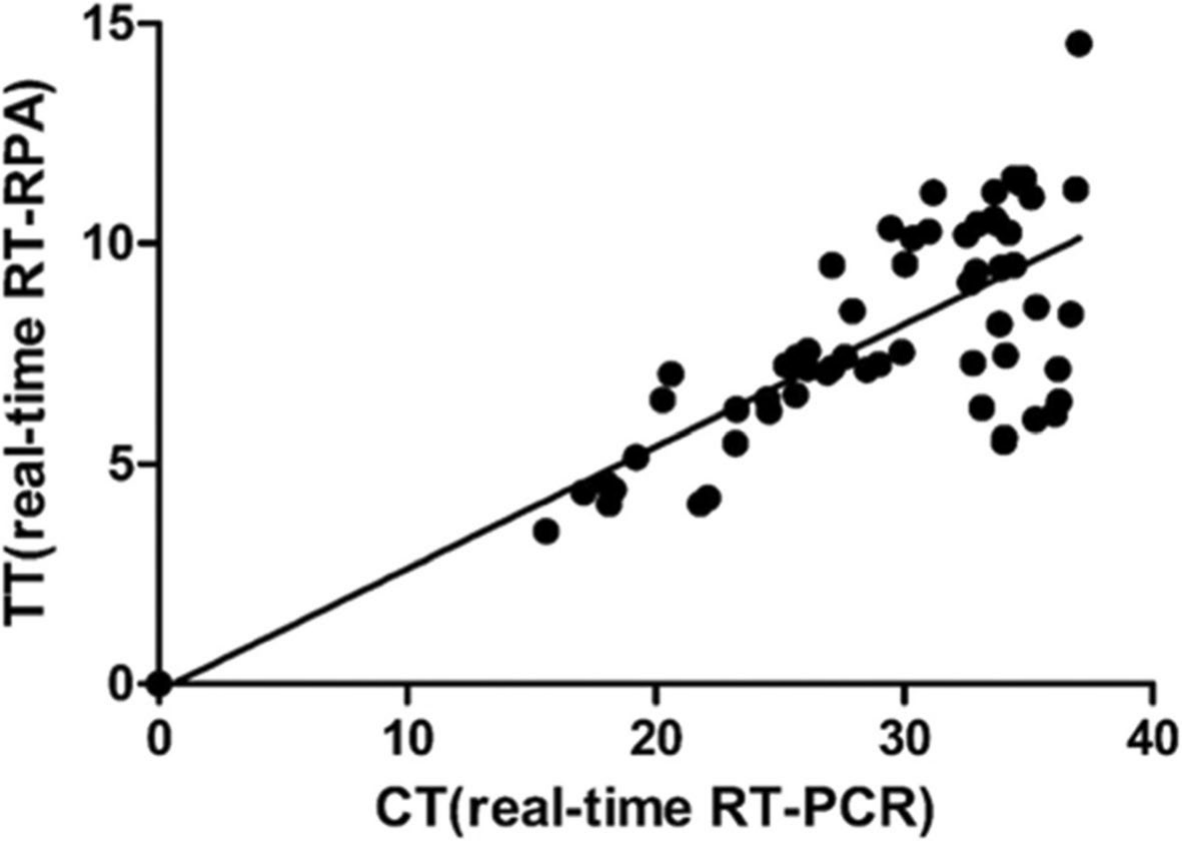
Comparison between performances of BRVA real-time RT-RPA and real-time RT-PCR on clinical samples. One hundred and thirty-four RNA extracts of the clinical samples were screened. The BRVA positive rate was 45.52% (61/134) and 46.27% (62/134) for real-time RT-RPA and real-time RT-PCR, respectively. Linear regression analysis of RT-RPA threshold time (TT) values (y axis) and real-time RT-PCR cycle threshold (Ct) values (x axis) were determined by Prism software, and R.^2^ value was 0.552

To evaluate the potential applicability of the RT-RPA assays, 134 cattle fecal samples had RNA extracted and were tested by the real-time RT-RPA, LFS RT-RPA and real-time RT-PCR. The BRVA positive rate were 45.52% (61/134), 46.27% (62/134) and 46.27% (62/134) in real-time RT-RPA, LFS RT-RPA and real-time RT-PCR, respectively (Table 1). Further analysis showed that the same detection results were obtained in LFS RT-RPA and real-time RT-PCR. The one sample negative in real-time RT-RPA while positive in other two assays had a Ct value of 38.12, indicating that the sample contained low amounts of BRVA RNA. Using the real-time RT-PCR assay as a reference, the diagnostic specificity of the real-time RT-RPA and LFS RT-RPA was 100%; the diagnostic sensitivity was 98.39% and 100%, and the kappa coefficients were 0.985 and 1.0, respectively (Table 2). The overall diagnostic agreement between the LFS RT-RPA and real-time RT-PCR was 100% (134/134). The overall diagnostic agreement between the real-time RT-RPA and real-time RT-PCR was 99.25% (133/134), and the threshold time (TT) and cycle threshold (Ct) values of the two assays were at an R^2^value of 0.552 (Fig. 4).

**Table 1:** BRVA detection results of the collected cattle fecal samples

Cattle months	Numbers	BRVA detection results (Positive, %)		
		Real-time RT-RPA	LFS RT-RPA	Real-time RT-PCR
≤ 3	78	55 (70.52%)	55 (70.52%)	55 (70.52%)
≥ 12	56	6 (10.71%)	7 (12.50%)	7 (12.50%)
Total	134	61 (45.52%)	62 (46.27%)	62 (46.27%)

**Table 2:** Diagnostic sensitivity, diagnostic specificity, predictive value, and kappa value of BRVA real-time RT-RPA, LFB RT-RPA and real-time RT-PCR assays

		Real-time RT-PCR		
		P	N	T
Real-time RT-RPA	P	61	0	61
	N	1	72	73
	T	62	72	134
		DSe:98.39%	DSp:100%	K:0.985
		PPV:100%	NPV:98.63%	
LFB RT-RPA	P	62	0	62
	N	0	72	72
	T	62	72	134
		DSe: 100%	DSp:100%	K:1.0
		PPV:100%	NPV: 100%	

*Note*: *P* Positive, *N* Negative, *T* Total, *DSe* Diagnostic sensitivity, *DSp* Diagnostic specificity, *K* Kappa coefficient value, *PPV* Positive predictive value, *NPV* Negative predictive value

## Discussion

BRVA is one of the most common pathogens causing calf diarrhea, which is widely prevalent worldwide and has seriously hampered the rapid development of the cattle industry [6, 10–12]. VP6 gene is the most conserved among the 11 gene segments of rotavirus and has become the preferred target gene for various molecular detection methods [16, 18, 19]. RPA is an emerging isothermal nucleic acid amplification technique [20], and could achieve exponential amplification of the target fragment in a short time (15 min ~ 30 min) at constant temperature (37 ℃ ~ 42 ℃), which has good operability and makes nucleic acid amplification detection under a variety of non-laboratory conditions possible, especially in remote areas such as grazing areas and farms. RPA had been widely used for rapid detection of a variety of pathogens [21–24], but no RPA method had been reported for BRVA detection according to our knowledge.

In this study, we designed specific RT-RPA primers and probes targeting the conserved region of VP6 gene, and established the real-time RT-RPA and LFS RT-RPA assays for specific detection of BRVA. Sequence comparison by blast search option of DNA STAR software revealed that the designed primers and probes showed 100% homology with most of the currently circulating BRVA strains deposited in GenBank, and had 1 ~ 6 nucleic acid mismatches with several strains. Several previous studies showed that RPA was tolerant to base mutations in primers and probes, and could tolerate 6 ~ 9 base mismatches without affecting the amplification effect [24]. In vitro transcribed VP6 gene was used as the standard RNA for sensitivity analysis in this study. The LOD of real-time RT-RPA was the same as that of real-time RT-PCR [18], which was 1.4 × 10^2^ copies/reaction, while the LOD of LFS RT-RPA was 1.4 × 10^1^ copies/reaction, which was 10 times higher than that of real-time RT-PCR. Moreover, both assays demonstrated good specificity, only BRVA but not the other common bovine diarrhea pathogens mentioned in the study was detected.

One hundred and thirty-four fecal samples from cattle with diarrhea were collected from 23 different farms in this study, and the detection rate of BRVA was 46.27% (62/134) for LFS RT-RPA and 45.52% (61/134) for real-time RT-RPA. BRVA mainly infected calves under 3 months [25, 26]. In this study, the BRVA positive rate in calves under 3 months was 70.52% (55/78), while the positive rate was only 12.50% (7/56) in adult cattle. Both the real-time RT-RPA and LFS RT-RPA assays showed the diagnostic specificity of 100% compared to real-time RT-PCR assay, and the diagnostic sensitivity of 98.39% and 100%, respectively. The performance of the RT-RPA assays was comparable to a previous described real-time RT-PCR [18], while the RT-RPA assays were faster to obtain the positive results.

RPA reagents are easy to store and transport, as they remain stable at room temperature and could be stored for about 20 days in high temperature environment (even at 45 °C) [27]. In addition, RPA is more tolerant to inhibitory compounds than PCR [24], which to some extent could explain the low correlation phenomenon we encountered in clinical sample detection (*r* = 0.552). The essence of RPA technology lies in the design of primers and probes. Currently, there is still no software specifically designed for RPA primers and probes, and researchers cannot judge the amplification performance of primers based on sequence alone. Therefore, candidate primers needed to be tested and screened to select the best primer–probe combination. Referring to the primer screening guide provided by TwistDx (http://www.twistdx.co.uk/), in this study, we designed 5 different primer pairs for the first time and verified the amplification effect by basic RT-RPA. Firstly, the RPA primer pairs producing only the specific targeted band were determined as the candidate. Subsequently, we shifted the sequences of the candidate primer pairs by 3 ~ 15 bases, and synthesized 3 forward and 5 reverse primers for a total of 15 combinations to choose the pairs producing the brightest targeted band in the basic RT-RPA. Finally, the determined primers from the second screening procedure were added or deleted by 1 ~ 3 bases, and 9 primer pairs and 1 exo probe were designed. The real-time RT-RPA assay was performed on the 9 combinations, and the best primer–probe combinations were screened based on amplification time and fluorescence intensity. It should be noted that the reagents of RPA is very viscous, so the template and the reagents need to be well mixed, otherwise it would affect the amplification efficiency [27].

The BRVA real-time RT-RPA was successfully performed in a portable tube scanner Genie III and LFS RT-RPA assay was successfully performed in a metal bath incubator. Both devices are small and portable, allowing the operator to carry them into the field for diagnostics. In addition, they could be recharged by batteries and could work all the day without recharging, making them suitable for extremely difficult field conditions. Interestingly, it has been recently reported [23] that amplification products (green fluorescence) from real-time RPA and LFS RPA analysis could be directly observed with the naked eye under a portable blue light imager with an excitation wave length of 480 nm. Although the results in our laboratory showed that the above described method could only work successfully for the samples containing high concentration targets, and it was difficult to observe a significant fluorescence signal for samples containing low concentration targets, it also provides a new possibility for the convenient detection of RPA products.

## Conclusions

The real-time RT-RPA and LFS RT-RPA assays were developed for reliable detection of BRVA in the fecal samples, and to our knowledge, this was the first report on the application of RPA in the BRVA detection. The developed RT-RPA assays were highly specific, sensitive, and easy to perform, which could be used as a routine analytical method for laboratory detection of BRVA and had great potential for application in the field (epidemic sites) such as farms and pasture areas.

## Methods

### Viral, bacteria strains and clinical samples

The common pathogens causing bovine diarrhea were used in this study. BRVA (BRVA, HB-LF/2021), Bovine viral diarrhea virus (BVDV, C24V), Bovine coronavirus (BCoV, HB-SJZ/2021), Bovine enterovirus (BEV, HB-BD/2021), Bovine kobuvirus (BKV, HB-ZJK/2022), Enterotoxigenic *Escherichia coli* (ETEC, CICC24190) and *Clostridium perfringens* (*C. perfringens*, CICC22949) were reserved in our laboratory.

One hundred and thirty-four fecal samples from the dairy cattle with diarrhea were collected from 23 cattle farms in 7 different regions of Hebei province and Datong of Shanxi province between May 2021 and March 2022. Seventy-eight samples were collected from calves under 3 months and the remaining 56 samples were collected from adult cattle (Table 3).

**Table 3:** Detailed information of the collected cattle fecal samples in this study

Locations	Cattle months	Numbers	Clinical symptoms
Baoding	≤3	18	water-like diarrhea
	≥ 12	17	diarrhea
Shijiazhuang	≤ 3	2	diarrhea
	≥ 12	24	diarrhea
Langfang	≤ 3	23	water-like diarrhea
Hengshui	≤ 3	7	water-like diarrhea
	≥ 12	7	diarrhea
Zhangjiakou	≤ 3	7	diarrhea
	≥ 12	6	diarrhea
Handan	≤ 3	10	water-like or bloody diarrhea
Datong, Shanxi	≤ 3	9	water-like diarrhea
Xingtai	≤ 3	2	water-like diarrhea
	≥ 12	2	diarrhea
Total		134	

### DNA/RNA extraction of the viruses, bacteria and the cattle fecal samples

BRVA, BVDV, BCoV, BEV and BKV viral RNA were extracted using TIANampViral RNA kit (Tiangen, Beijing, China), and ETEC and *C*. *perfringens* bacterial genomic DNA were extracted using the TIANamp bacterial DNA kit (Tiangen, Beijing, China), which were all performed according to the manufacturer’s instructions. The fecal samples were homogenized with phosphate buffered saline (PBS, pH7.4) as a 10% (w/v) suspension and centrifuged for 5 min at 6000 g at 4 °C. Two hundreds microliter of the supernatant was used for viral RNA extraction using the TIANLONG Magnetic Viral DNA/RNA kit (Tianlong, XiAn, China) according to the manufacturer’s instructions. The viral RNA was extracted using the Automatic Nucleic Acid Extraction Instrument (np968-c, Tianlong, XiAn, China). Viral RNA and bacteria DNA were quantified using ND-2000c spectrophotometer (NanoDrop, Wilmington, USA). All RNA and DNA templates were used immediately or stored at − 80 °C.

### Generation of the BRVA RNA standard

The BRVA RNA standard for the RT-RPA assays was generated as previously described [28]. The BRVA viral RNA was reverse transcribed using the PrimeScript™ 1st strand cDNA Synthesis Kit (TaKaRa, Dalian, China) and the generated cDNA was used as template in the following PCR assay. The RT-PCR primers (BRVA-VP6-F and BRVA-VP6-R) were designed based on the conserved sequence of VP6 gene (Table 4). The PCR reaction was consisted of 12.5 μL of 2 × Go Taq® Green Master Mix (Promega, Madison, USA), 1 μL of cDNA, 1.0 μL of each of primers (10 μmol/L) and 9.5 μL of Nuclease free water. The reaction was performed as following: 95 °C for 5 min, followed by 40 cycles of 95 °C for 30 s, 56 °C for 30 s and 72 °C for 90 s, and a final extension step at 72 °C for 10 min by using Gradient PCR instruments (Applied Biosystems, Foster City, California). The 1177 bp PCR product were purified using the TIANgel Midi Purification Kit (Tiangen, Beijing, China), ligated into a pGEM-T Easy vector (Promega, Madison, USA) and transformed into *E. coli* DH5α. The in vitro transcribed BRVA standard RNA was produced with RiboMAX Large Scale RNA Production System-T7 (Promega, Madison, USA), quantified using ND-2000c and the copy number of RNA molecules was calculated by the formula: Amount (copies/μL) = [RNA concentration (g/μL)/(transcript length in nucleotides × 340)] × 6.02 × 10^23^. The in vitro transcribed RNA was diluted in tenfold serial dilutions to achieve RNA concentrations ranging from 1.4 × 10^5^ to 1.4 × 10^0^ copies/μL and stored at − 80 °C, which were used as the standard RNA in the following study.

**Table 4:** Sequences of the primers and probes for BRVA RT-PCR, real-time RT-RPA, LFS RT-RPA and real-time RT-PCR assays

Assays	Primers and probes	Sequence (5´-3´)	Amplicon size (bp)	Reference
RT-PCR	VP6-F	ATGGATGTCCTGTACTCCTTGTC	1177	This study
	VP6-R	TTCTAATGGAAGCCACTGTAAATAC		
real-time RT-RPA	BRVA-exo-F	GTAGATAATGTATGTATGGACGAGATGGTT	192	This study
	BRVA-exo-R	TGGCTTATGAAATGTGAAACCCGTTCTTTG		
	BRVA-exo-P	TTGCACCACAATCAGATTCACTCAGAAAGT(FAM-dT)(THF)(BHQ1-dT)CAGGTATTAAATTC-C3-spacer		
LFS RT-RPA	BRVA-nfo-F	GTAGATAATGTATGTATGGACGAGATGGTT	192	This study
	BRVA-nfo-R	Biotin-TGGCTTATGAAATGTGAAACCCGTT CTTTG		
	BRVA -nfo-P	FAM-TTGCACCACAATCAGATTCACTCAGAAAGTT(THF)TCAGGTATTAAATTC-C3-spacer		
real-time RT-PCR	BRVA-qPCR-F	ACTCCAATGTAAGTGATCTAATTC	138	[18]
	BRVA-qPCR -R	GAGTTGTTCCAAGTAATCCAAA		
	BRVA-qPCR -P	FAM-ACCAATTCCTCCAGTTTGGAAYTCATTYCC-BHQ1		

### Primers and probes of the RT-RPA assays

The VP6 gene is highly conserved among the different BRVA strains, so it is used as the target for RT-RPA assays. The RPA primers, exo probe and nfo probe were designed based on the conserved region of VP6 gene, which were collected from the different BRVA strains deposited in GenBank (Accession numbers: MN047454.1; MT240631.1; MK250428.1; MK638874.1; MT240629.1; X53667.1; LC336590.1; AF411322.2; HM988974.1; K02254.1). The RPA primers and probes were synthesized by Generay (Shanghai, China), and the information of them was presented in Table 4.

### Real-time RT-RPA assay for BRVA

Real-time RT-RPA assay for BRVA was performed using a ZC BioScience™ exo kit (ZC BioScience, Hangzhou, China) as previously described with minor modifications [22]. The reaction volume was 50 μL including 25 μL of Buffer A (rehydration buffer), 2.0 μL of each RPA primer (BRVA-exo-F and BRVA-exo-R, 10 μmol/L), 0.6 μL of exo probe (BRVA-exo-P, 10 μmol/L), 2.5 μL of Buffer B (magnesium acetate, 280 mmol/L) and a total of 17.9 μL of Nuclease free water and RNA template. Additionally, 1 μL of BRVA standard RNA was used for the specificity and sensitivity analysis, while 3 μL of sample RNA was used for the clinical sample diagnosis. All reagents except for the Buffer B (magnesium acetate) were distributed into each 0.2 ml freeze-dried reaction tube containing a dried enzyme pellet. Subsequently, magnesium acetate was pipetted into the tube lids, then the lids were closed carefully and the magnesium acetate was centrifuged into the rehydrated material using a minispin centrifuge. The reaction tubes were vortexed briefly and spun down once again, and the tubes were immediately placed in the Genie III scanner device (OptiGene Limited, West Sussex, UK) to start the reaction at 42 °C for 20 min.

### LFS RT-RPA assay for BRVA

LFS RT-RPA assays for BRVA were performed using a GenDx ERA Kit (GenDx Biotech, Suzhou, China) as previously described with minor modifications [22]. The reaction volume was 50 μL including 20 μL of Buffer A (rehydration buffer), 2.1 μL of each RPA primer (BRVA-nfo-F and BRVA-nfo-R, 10 μmol/L), 0.6 μL of nfo probe (BRVA-nfo-P, 10 μmol/L), 2.0 μL of Buffer B (magnesium acetate, 280 mmol/L) and a total of 23.2 μL of Nuclease free water and RNA template. Additionally, 1 μL of BRVA standard RNA was used for the specificity and sensitivity analysis, while 3 μL of sample RNA was used for the clinical sample diagnosis. The procedure for adding reagents and samples was the same as the procedure for real-time RT-RPA, then the reaction tubes were incubated at optimal temperature for suitable reaction time in a thermostatic metal bath. The subsequent RT-RPA products were analyzed using the lateral flow strips (GenDx Biotech, Suzhou, China) according to the manufacturer’s instructions.

### Optimization of incubation temperature and time of LFS RT-RPA

To determine the optimal incubation temperature, the LFS RT-RPA reactions were carried out on a Gradient PCR instrument (Applied Biosystems, Foster City, California) set at 39, 40, 41, 42, 43, 44 and 45 °C for 20 min using 1.4 × 10^2^ copies of standard RNA as templates. The amplification assays were performed in parallel for each temperature. The subsequent RT-RPA products were analyzed by the lateral flow strip analysis. To define the optimal incubation time, the reactions were performed at the specified optimal temperature for 10, 15, 20 and 30 min using 1.4 × 10^2^ copies standard RNA as templates followed by the lateral flow strip analysis. The amplification assays with 4 different incubation time were performed independently and repeated 3 times.

### Analytical specificity and sensitivity analysis

The real-time RT-RPA and LFS RT-RPA assays were performed with the nucleic acids of a panel of pathogens including BRVA, BVDV, BCoV, BEV, BKV, ETEC and *C*. *perfringens*, which are important pathogens causing diarrhea in cattle. In the assay, Nuclease free water was used as the non-template control. Three independent reactions were performed.

The constructed BRVA RNA standards ranging from 1.4 × 10^5^ and 1.4 × 10^0^ copies/μL were used in the analytical sensitivity analysis of the real-time RT-RPA and LFS RT-RPA assays, in which Nuclease free water was used as the non-template control. One microliter of each dilution was used as template in both RT-RPA assays, and the limit of detection (LOD) was determined to be the highest dilution of the viral RNA detected by the assays. Eight independent real-time RT-RPA and 5 independent LFS RT-RPA repeats were performed.

### Real-time RT-PCR assay

Real-time RT-PCR assay was used to detect the BRVA in the cattle fecal samples using primers and probe from a previously published protocol [18]. The reaction volume was 25 μL, which included 12.5 μL of 2 × PerfectStart™ Probe One-Step qPCR SuperMix (TransGen Biotech, Beijing, China), 0.5 μL of TransScript^@^ Probe One-Step RT/RI Enzyme Mix, 0.5 μL of each primer and probe (10 μmol/L), 3 μL of RNA template and 7.5 μL of Nuclease free water. The reaction condition was 94 °C for 5 min, 94 °C for 30 s, 40 cycles of 94 °C for 5 s and 60 °C for 30 s. Real-time RT-PCR was performed using ABI Quant Studio 5 (Applied Biosystems, Foster City, California).

### Validation of the developed RT-RPA assays with the cattle fecal samples

The applicability of the BRVA real-time RT-RPA and LFS RT-RPA assays in the clinical diagnosis was validated using the RNA extracted from 134 cattle fecal samples as template. Then, the results were compared with those obtained with a real-time RT-PCR described previously [18], which was run in parallel for the above clinical samples.

## Data Availability

The dataset analyzed during the current study is available from the corresponding author on reasonable request. The nucleotide sequences under the relevant accession numbers (MN047454.1; MT240631.1; MK250428.1; MK638874.1; MT240629.1; X53667.1; LC336590.1; AF411322.2; HM988974.1; K02254.1) analyzed during the current study are available in the GenBank repository, https://www.ncbi.nlm.nih.gov/nucleotide/.

## Abbreviations

BRVA: Bovine rotavirus A
BRV: Bovine rotavirus
BVDV: Bovine viral diarrhea virus
BCoV: Bovine coronavirus
BEV: Bovine enterovirus
BKV: Bovine kobuvirus
CT: Cycle threshold
C.perfringens: Clostridium perfringens
DSp: Diagnostic specificity
DSe: Diagnostic sensitivity
ETEC: Enterotoxigenic Escherichia coli
E. coli: Escherichia coli
K: Kappa coefficient value
LFS: Lateral flow strip
LOD: Limit of detection
NPV: Negative predictive value
PCR: Polymerase chain reaction
PPV: Positive predictive value
PBS: Phosphate buffered saline
RPA: Recombinase polymerase amplification
RT-RPA: Reverse transcription recombinase polymerase amplification
RT-LAMP: Reverse transcription loop-mediated isothermal amplification
RT-PCR: Reverse transcription polymerase chain reaction
RVA: Rotavirus
SSB: Single-stranded DNA-binding protein
TT: Threshold time
